# Competition in the Brain. The Contribution of EEG and fNIRS Modulation and Personality Effects in Social Ranking

**DOI:** 10.3389/fpsyg.2016.01587

**Published:** 2016-10-13

**Authors:** Michela Balconi, Maria E. Vanutelli

**Affiliations:** ^1^Research Unit in Affective and Social Neuroscience, Catholic University of Milan, MilanItaly; ^2^Department of Psychology, Catholic University of Milan, MilanItaly

**Keywords:** social ranking perception, alpha oscillation, fNIRS, BAS, left lateralization

## Abstract

In the present study, the social ranking perception in competition was explored. Brain response (alpha band oscillations, EEG; hemodynamic activity, O2Hb), as well as self-perception of social ranking, cognitive performance, and personality trait (Behavioral Activation System, BAS) were considered during a competitive joint-action. Subjects were required to develop a strategy to obtain a better outcome than a competitor (C) (in term of error rate, and response time, RT). A pre-feedback (without a specific feedback on the performance) and a post-feedback condition (which reinforced the improved performance) were provided. It was found that higher-BAS participants responded in greater measure to perceived higher cognitive performance (post-feedback condition), with increased left prefrontal activity, higher ranking perception, and a better real performance (reduced RTs). These results were explained in term of increased sense of self-efficacy and social position, probably based on higher-BAS sensitivity to reinforcing conditions. In addition, the hemispheric effect in favor of the left side characterized the competitive behavior, showing an imbalance for high-BAS in comparison to low-BAS in the case of a rewarding (post-feedback) context. Therefore, the present results confirmed the significance of BAS in modulating brain responsiveness, self-perceived social position, and real performance during an interpersonal competitive action which is considered highly relevant for social status.

## Introduction

The effect of a competitive task in the brain was recently explored by different perspectives (Cui et al., 2015; Liu et al., 2015). There is a broad agreement that competition during an interpersonal performance essentially implies a process of social comparison in addition to the explicit evaluation of subjective performance. That is, when competition induces a “social outcome” and a potential improving in social hierarchy, it may produce multiple effects directly related to the “social” significance of the competition and to self-perception within an interpersonal context. That is the comparison between my own and others’ outcomes on a specific interpersonal task may or may not improve my rank perception and social status representation in term of efficacy, taking into account the existing interpersonal condition. Indeed, in human interpersonal dynamics, social hierarchies can be established along various dimensions: we can be socially ranked according to abilities or skills, as well as to economic, physical, and professional standing.

Conversely, previous research also suggested an important role for social interactions and self-perception in achieving accurate self-knowledge and self-improvement, particularly in response to performance-related social comparisons. It was shown, in fact, that social status perception reciprocally affects performance on tasks that involve comparing our own performance with that of others (Munafò et al., 2005): in other words, the specific analysis of the social status in the context of performance-based feedback may act on the real subjective performance by improving or decreasing the subject’s cognitive behavior (Munafò et al., 2005). Some previous studies explored the effect of competition on self-perception, on the efficacy in social interaction, and on the social-ranking within the social hierarchy. It was found that competition may increase the effective subjective performance and perception of higher social ranking, but it contemporarily induces a decreased sense of in-group and make the sense of social membership more weak (Goldman et al., 1977). That is, the subject pays for his/her better performance in terms of “being less socially part of.”

Therefore, competition is a social-evaluative phenomenon and such competitive situations can increase the level of cognitive demand on the performer beyond what is required to simply execute the task. The increase in such demand may explain, in part, how competition influences the quality of performance (with a better or worse effect) and brain responsiveness due to the attendant alterations of the performer’s mental state and the underlying neural processes (Rietschel et al., 2011).

It was also shown that an extended neural circuit linking limbic, prefrontal, and striatal structures may reflect the emotional, cognitive, and behavioral components of rank-related social interactions (Levitan et al., 2009). Recent research examining the structure and function of brain areas associated to social perception, social efficacy and social ranking offered preliminary support for these neural mechanisms of human social system. Dorsal (DLPFC) and ventral (VLPFC) portions of lateral prefrontal cortex (PFC) are generally recruited during social status inference (Chiao et al., 2009b; Balconi and Pagani, 2014, 2015). The activation of DLPFC and VLPFC during the observation of social interactions and social status implications probably reflects the recruitment of brain regions that can exert top-down control over specific processes, such as emotional responses to social hierarchy, to orchestrate a socially appropriate status response (Marsh et al., 2009). Indeed, these brain regions are typically associated to socio-emotional regulating responses and behavioral inhibition.

More specifically, the perception of competition influences the internal evaluation and may manifest as an increase in cerebral cortical activation in some prefrontal areas, and affect the performance outcomes. Cui et al. (2015) have measured pairs of participants’ prefrontal activations during concurrent cooperation and competition using near-infrared spectroscopy (NIRS). The participant pairs showed increased inter-brain synchronization in their right superior frontal cortices during cooperation (but not during competition), due to the requirements of modeling the behaviors of others in the cooperative interactions. Moreover, it has been shown that the processing load associated to the competitive condition resulted in heightened cortical activity, as measured by high-alpha electroencephalographic (EEG) power, across all examined brain regions. As such, competition imposed an increase in cognitive load. In addition, the increase in cortico-cortical communication was robust, involving heightened communication between all non-motor regions with the strategy planning region, that is specifically in the prefrontal areas.

However, it remains to be answered whether and how an increase in cerebral cortical activity is specifically promoted by competition-induced social evaluation when the cognitive performance is artificially manipulated. Indeed no previous research, to date, has manipulated the social environment via direct competition to assess this possibility. Thus, taking into account previous research, two relevant aspects were underestimated and, in our opinion, should be deeply considered to evaluate the competition effect on social self-perception and cognitive outcomes: firstly, the presence of an inter-subjective real interaction where the co-partner is present and actively implicated in the competitive exchange (Montague, 2002); secondly, some personality components related to motivational and emotional level, such as approach/withdrawal attitude to emotions (Gray, 1990; Balconi et al., 2012).

About the first aspect, in the present research the subject’s performance on a cognitive task and his/her related social ranking were artificially manipulated in a dyadic vis-à-vis competition which stressed the necessity to win and to obtain a better score than the partner, therefore in interaction with a competitor (C). Indeed, in contrast to previous studies (Zink et al., 2008), we included a more realistic ecologic task, where subjects were required to constantly compare their performance with that of the other subject. Specifically, an online comparison with C was performed so that the dynamic modifications of the subjects’ performance were constantly compared. This aspect strongly modulated the subject’s perceived status in terms of performance (“your performance is better than…”) and, based on this comparison, we tested the effect on the subject’s own status modification related to C status.

About the second aspect, we supposed that the way individuals judge their social ranking positions partially depends on some personality factors, such as the degree to which their own behavior is balanced between “approaching” in response to rewards (to be more responsive to winning) and non-punishments, or “withdrawing” from non-reward and punishments (being more sensitive to losing). These emotional and motivational components appear to be highly relevant with respect to self-perception in social contexts and social ranking. Indeed recent research found that motivations and emotions are able to manipulate the perception of social hierarchies by inducing more positive versus negative predispositions in social relationships (Marsh et al., 2009). Specifically, it was previously found that subjects with a higher-BAS (the Behavioral Activation System; Gray, 1994) were more likely to relate to the dominant character in a dyadic interaction, which was found to induce a positive effect and a better representation of their own social status, while those with a higher BIS (the Behavioral Inhibition System; Farrow et al., 2001) were more inclined to relate to a submissive character and to produce more negative representations of their own social status (Demaree, 2005). Moreover, in previous research a significant BAS effect was found in distinguishing social hierarchy (Demaree et al., 2005; Balconi and Pagani, 2014, 2015). More generally, the BAS system is conceptualized as a motivational system that is sensitive to signals of reward, non-punishment, and it is responsible for both approach and active behaviors. Emotions associated with these behaviors generally induce the subject to positively approach to interpersonal situation that have generated the emotional response. In addition BAS has been associated to feelings of optimism and dominance: people with highly sensitive BAS may respond in great measure to approach-related emotional contexts, that allow the subject to have a favorable and dominant behavior toward the environment (Davidson et al., 1990; Tomarken et al., 1992; Gable et al., 2000; Gray and McNaughton, 2000; Balconi and Mazza, 2009, 2010; Balconi et al., 2009a,b, 2012).

About BIS/BAS cortical correlates, they appear to be lateralized and they are viewed as mutually inhibitory: respectively, the left PFC was shown to support the approach-related motivations and emotions, whereas the right PFC was found to be involved in withdrawal-related motivations and emotions (Bechara et al., 1999; Bechara and Martin, 2004; Balconi and Mazza, 2010; Balconi et al., 2012). Therefore, the role of these two antithetic prefrontal systems, on one hand, and that of the frontal (PFC) “social” brain circuit was supposed to be able to elucidate the self-perception of social hierarchy. To explore the cortical impact in concomitance with the BIS/BAS dichotomy, the EEG and hemodynamic activity was monitored during the competitive dyadic task.

Indeed, firstly the modulation of EEG brain oscillations was considered as a valid measure of brain activation, and it has often been applied to describe distinct responsiveness by the two hemispheres to different emotional and social conditions (Sutton and Davidson, 1997; Balconi and Mazza, 2009; Balconi et al., 2012; Balconi and Vanutelli, 2015). In fact a reduction of alpha power (increased cortical activity) in the left frontal brain was found in response to approach attitude (Balconi and Mazza, 2010; Balconi et al., 2011), whereas withdrawal conditions induced reduction in alpha power in the right frontal brain (Davidson, 1992, 2004; Harmon-Jones, 2004; Balconi et al., 2009a,b). Secondly, although some studies have provided functional images of activated brain areas in relation to social ranking (Chiao et al., 2009b; Freeman et al., 2009; Marsh et al., 2009), they have scarcely addressed the temporal course of such activation. The classical imaging measures (i.e., functional Magnetic Resonance, fMRI) do not seem to completely describe in depth the nature of the dynamic social processes. Due to its fast temporal evolution and its representation and integration among complex and extended neural areas, social interactions should preferably be examined by means of imaging methods that offer good resolution in both temporal and spatial domains. Temporal resolution of NIRS is high enough for measuring event-related hemodynamic responses (Elwell et al., 1993). Finally, combined EEG/NIRS measurements allow for the complementary examination of neural as well as hemodynamic aspects of brain activation in social dynamics (Biallas et al., 2012; Balconi et al., 2015).

Therefore, the aim of the present study was to investigate the neurophysiological bases of social ranking perception underlying the execution of a competitive joint-action by using both EEG and fNIRS acquisition. Based on our hypotheses, the observed performance and the external feedback from one hand, and the personality components (BIS/BAS) from the other hand, may affect the self-perception of social position and hierarchy, and they effectively may modulate our cognitive performance in social contexts. That is, the perceived effectiveness of our behavior in term of performance during a competitive task and specific BAS components positively guide self-perception of our position within the social ranking and consequently these mechanisms may impact on the real cognitive outcomes (improved performance). Concerning the cognitive performance, consistent better performance should be found for higher-BAS trait when perceiving an improved ranking, as an effect of more reinforcing and rewarding outcomes. That is, the “improving performance effect” should be more significant in high-BAS as a concomitant result of perceived dominance and rewarding situations, which high-BAS judge as positive in greater measure.

The cortical correlates of these cognitive and social mechanisms are supposed to be related to the prefrontal areas, with a specific contribution of the left and right hemisphere. Therefore it is crucial to consider the implication of the PFC and a possible lateralization effect in response to social ranking perception, in combination with personality components (Hall et al., 2005; Chiao et al., 2009a; Balconi and Pagani, 2014, 2015). We may suppose that, based on the lateralized approach/withdrawal model, there could be different contributions of the left and right hemispheres on self-perception of social ranking. Based on these previous results, it should be plausible that the hemispheric “competition” between the left and right sides would characterize social hierarchy behavior, showing a greater approach attitude and dominance in higher competitive condition with an imbalance in favor of the left hemisphere. Specifically, we supposed that higher-BAS (high-BAS) more than higher BIS participants (high-BIS) may respond in greater measure to increased outcomes and increased self-perception of higher social ranking due to the rewarding effect of higher dominance conditions. Therefore, decreased alpha activity (i.e., increased brain responsiveness) for EEG and increased oxygenated hemoglobin (O2Hb) for fNIRS should be found, respectively, for higher-BAS in the frontal left brain area when they perceived increased performance.

Finally, the three levels of social ranking perception, personality components, and cognitive performance should be consonant, since we expected a correlated increased self-perception of social position and a better performance in relation to higher-BAS, with a concomitant higher activation of left prefrontal areas.

## Materials and Methods

### Subjects

Twenty-four undergraduate students, 10 males and 14 females, took part to the experiment (*M* = 21.06, *SD* = 3.24). The participants were all right-handed and presented normal or corrected-to-normal visual acuity. Exclusion criteria were history of psychopathology (Beck Depression Inventory, BDI-II, Beck et al., 1996; State-Trait-Anxiety-Inventory, STAI, Spielberger et al., 1970) for the subjects and immediate family. No neurological or psychiatric pathologies were observed. Subjects gave informed written consent to participate in the study and no payment was provided for subjects’ performance. The research was approved by the local ethics committee of the Department of Psychology, Catholic University of Milan.

### Procedure

Subjects were seated comfortably in a moderately darkened room with a monitor screen positioned approximately 60 cm in front of their eyes. A modified version of Balconi and Pagani (2014) was used in the present experiment. Participants were told that some cognitive attentional measures were used to evaluate the subjective skills and, to reinforce their motivation, that these measures were usually applied as a screening to test their future professional career success (teamwork capabilities). In addition, the competitive nature of the task was stressed: participants were told that the scoring was based on the capacity to produce a better performance than the C, in term of accuracy (number of errors: Error Rate, ER) and response times (RTs). They were seated side-by-side, but separated by a black screen in a way that they couldn’t see each other.

**FIGURE 1:**
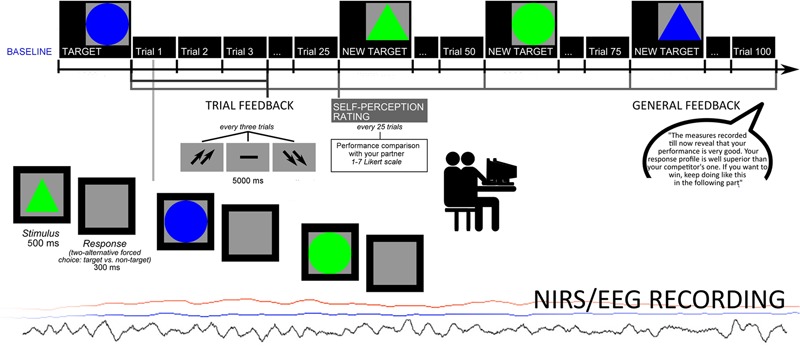
**Experimental procedure which represents setting, task, and measure acquisition**.

The cognitive task consisted in a sustained selective attention task. Participants were required to select a target stimulus between non-targets, based on four different options of shape/color: the stimuli might interchangeably be a triangle or a circle, colored red or green. They were required to distinguish between target/non-target by focusing attention on each stimulus. The target was displayed on the video (indicated as the target for selection) and the successive stimuli were presented one after another. The target stimulus features changed every 25 trials. The subjects were instructed to make a two-alternative forced-choice response by pressing a left/right button. Each stimulus was presented for 500 ms, with a 300 ms inter-stimulus interval (ISI). After each trial, composed by three stimuli, subjects received a feedback, after 5000 ms, signaled by two up-arrows (high score); a dash (mean performance); or two down-arrows (low score). This feedback remained for 5000 ms. After the feedback, an inter-trial interval (ITI) occurred for other 5000 ms. The task was composed by two sessions: the first which did not include a specific general feedback to performance (four blocks before the feedback, 100 trials), and a second one which included a specific positive feedback to performance (four blocks with the feedback, 100 trials; **Figure 1**). Halfway, in fact, participants received a general evaluation of their performance. Actually, both trial-feedbacks and the general-feedback were artificially managed. The feedback order (two sessions) was counterbalanced across subjects. For what concerns the general feedback, participants were told that they had an outcome “well above” their competitor’s one and were encouraged to maintain their performance level, during the second part of the experiment (“The measures recorded till now reveal that your performance is very good. Your response profile is well superior than your competitor’s one. If you want to win, keep going like this in the following part”). Across the task, after an initial mean performance, subjects were constantly reinforced about their good performance by presenting the up-arrows in 70% of cases, while the dash or the down-arrows appeared only in 30% of cases (mainly at the beginning of the task) to make the task more credible and plausible. In addition, after each block of 25 trials, subjects were required to evaluate their performance and efficacy in term of their ranking on a 7-point Likert scale (from one = most decreased ranking due to performance, to seven = most improved ranking due to performance). Finally a post-session questionnaire explored the following aspects: degree of their engagement in the task; trust in the received feedback; relevance of task for their social status perception; perceived improving of ranking position. The data showed that participants were strongly engaged in the task (94% were strongly engaged); trusted in external feedback (95%); considered the task as relevant for social status (94%); improved their ranking position during the task (96%).

### BAS Scoring

Behavioral Activation System scores were calculated by using the Italian version (Leone et al., 2002) of Carver and White Questionnaire (Carver and White, 1994). It included 24 items (20 score-items and four fillers, each measured on 4-point Likert scale), and two total scores for BIS (range = 7–28; items 7) and BAS (range = 13–52; items 13). BAS also includes three subscales (Reward: five items; Drive: four items; Fun Seeking: four items). The questionnaire was submitted to the participants after completing the experimental phase. The total scores for each scale were, respectively: BAS: *M* = 47.90 (*SD* = 3.91); Reward: *M* = 23.76 (*SD* = 2.88); Drive: *M* = 12.98 (SD = 3.15); Fun Seeking: *M* = 13.09 (*SD* = 2.11). Cronbach’s alpha was calculated for BAS (0.96) and for each BAS subscale (Reward = 0.87; Drive = 0.88, and Fun Seeking = 0.89). Based on these ratings we created two sub-groups: high-BAS and low-BAS subjects. The first group included subjects with high-BAS scoring (more than 48, mean + 1 SD, *N* = 10); the second group included subjects with low BAS scoring (less than 44, mean - 1 SD, *N* = 14). Since BIS and BAS were orthogonally distributed and systematically participants higher in BAS were lower in BIS, BIS was not used in this phase of research. Only one subject was removed from the final analysis since he showed a mixed-profile (both high-BAS and high-BIS score).

About gender distribution, for both high-BAS and low-BAS groups there was an equal distribution (respectively, high-BAS: female = 6, male = 4; low-BAS female = 8, male = 6) of genders.

### EEG Analysis

Electroencephalographic recordings were performed with two 16-channel portable EEG-System (V-AMP: Brain Products, München. Truscan: Deymed Diagnostic, Hronov). An ElectroCap with Ag/AgCl electrodes was used to record EEG from active scalp sites referred to the earlobes (10/5 system of electrode placement; Oostenveld and Praamstra, 2001). Data were acquired using a sampling rate of 500 Hz, with a frequency band of 0.01–40 Hz. An off-line common average reference was successively computed to limit the problems associated with the signal-to-noise ratio (Nunez and Srinivasan, 2006). One EOG electrode was placed on the outer canthi to detect eye movements. The impedance of the recording electrodes was monitored for each subject prior to data collection and was always below 5 kΩ. The signal was visually scored, and portion of the data that contained artifacts were removed to increase specificity. Blinks were also visually monitored. Ocular artifacts (eye movements and blinks) were corrected using an eye-movement correction algorithm that employs a regression analysis in combination with artefact averaging (Sapolsky, 2004). After performing EOG correction and visual inspection, only artifact-free trials were considered (rejected epochs, 3%).

The digital EEG data were band-pass filtered in the frequency band 8–12 Hz (high- and low-alpha; band-pass filtering 96 dB/octave rolloff, warm-up filter left and right to 100 ms). To obtain a signal proportion to the power of the EEG frequency band, the filtered signal samples (epoch 1000 ms) were squared (Pfurtscheller, 1992). An average absolute power value for each experimental condition was calculated. An average of the pre-experimental absolute power (-200 ms) was used to determine the individual power during no stimulation.

**FIGURE 2:**
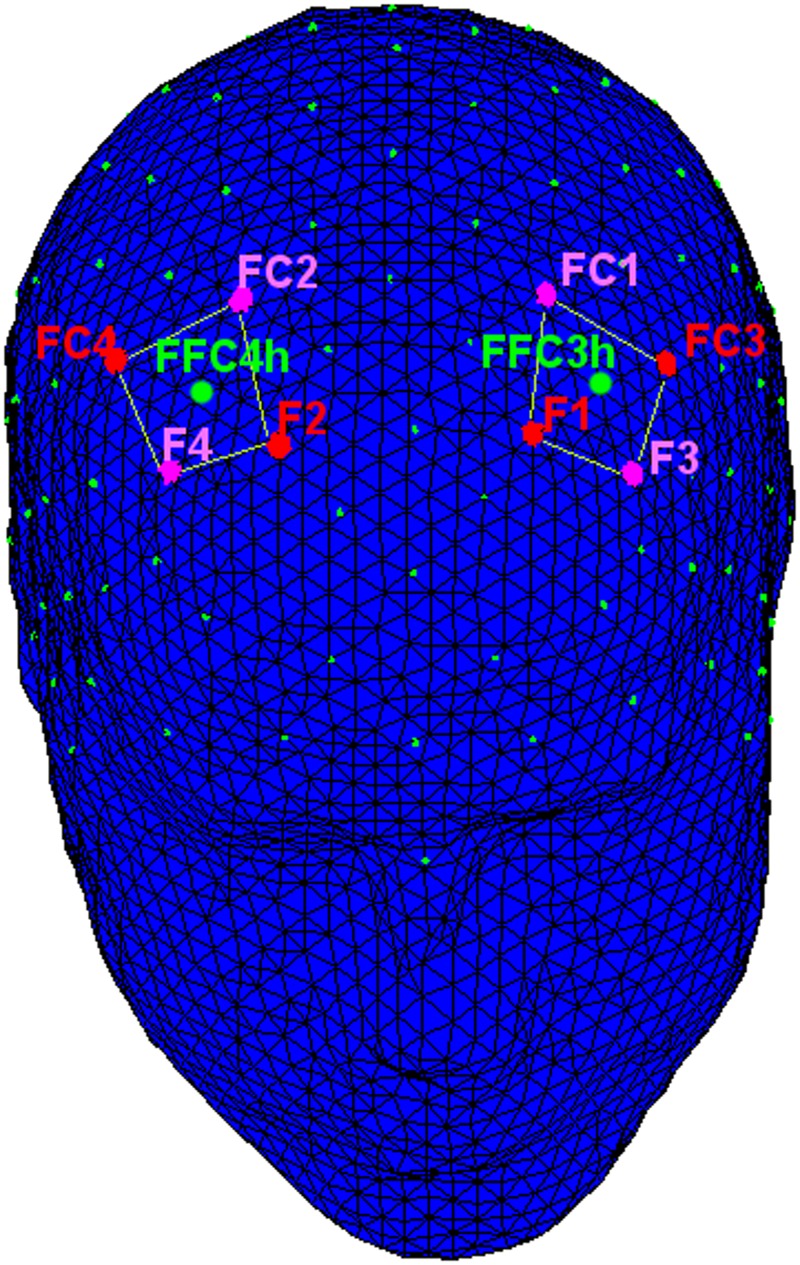
**The location of the measurement near-infrared spectroscopy (NIRS)/electroencephalographic (EEG) channels.** NIRS: The emitters were placed on positions FC3–FC4 and F1–F2 (red dots), while detectors were placed on FC1–FC2 and F3–F4 (pink dots). EEG: left and right frontal (green dots: FFC3h and FFC4h) alpha power activity was considered.

The following channels were acquired. For the statistical analysis left and right frontal (FFC3h, FFC4h) alpha power activity was considered (**Figure 2**).

### fNIRS

**FIGURE 3:**
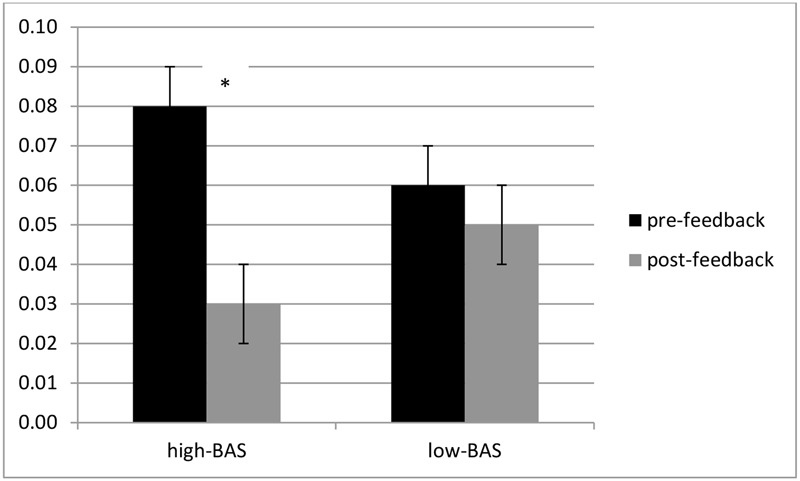
**Error rates modulation as a function of Behavioral Activation System (BAS) and Condition.** High-BAS showed an increased ER in post-feedback compared to pre-feedback condition while they did not differ from low-BAS in pre- or post-feedback. Also no significant differences were found for low-BAS between pre- and post-feedback. Asterisk indicates significant differences.

fNIRS measurements were conducted with the NIRScout System (NIRx Medical Technologies, LLC, Los Angeles, CA, USA) using a 8-channel array of optodes (four light sources/emitters and four detectors) covering the prefrontal area. Emitters were placed on positions FC3–FC4 and F1–F2, while detectors were placed on FC1–FC2 and F3–F4 (**Figure 3**). Emitter-detector distance was 30 mm for contiguous optodes and near-infrared light of two wavelengths (760 and 850 nm) were used. NIRS optodes were attached to the subject’s head using a NIRS-EEG compatible cup, with respect to the international 10/5 system.

With NIRStar Acquisition Software, changes in the concentration of oxygenated (O2Hb) and deoxygenated hemoglobin (HHb) were recorded from a 120 s starting baseline. Signals obtained from the eight NIRS channels were measured with a sampling rate of 6.25 Hz, and analyzed and transformed according to their wavelength and location, resulting in values of the changes in the concentration of oxygenated and deoxygenated hemoglobin for each channel. Hemoglobin quantity is scaled in mmol^∗^mm, implying that all concentration changes depend on the path length of the NIR light in the brain.

The raw data of O2Hb and HHb from individual channels were digitally band-pass filtered at 0.01–0.3 Hz. Successively, the mean concentration of each channel within a subject was calculated by averaging data across the trials from the trial onset for 5 s. Based on the mean concentrations in the time series, we calculated the effect size in every condition for each channel within a subject. The effect sizes (Cohen’s d) were calculated as the difference of the means of the baseline and trial divided by the standard deviation (SD) of the baseline: *d* = (m1 - m2)/s. Accordingly, m1 and m2 are the mean concentration values during the baseline and trial, and s means the SD of the baseline. The mean concentration value of 5 s immediately before the trial was used as event-related baseline. Then, the effect sizes obtained from the eight channels were averaged in order to increase the signal-to-noise ratio. Although the raw data of NIRS were originally relative values and could not be averaged directly across subjects or channels, the normalized data such as the effect size could be averaged regardless of the unit (Schroeter et al., 2003; Matsuda and Hiraki, 2006; Shimada and Hiraki, 2006). In fact, the effect size is not affected by differential pathlength factor (DPF; Schroeter et al., 2003).

## Results

Four sets of analyses were performed with respect to behavioral (ER; RTs; self-perception ranking) and neurophysiological (alpha band and O2Hb measures) measures. Repeated measure ANOVAs were applied to these dependent measures with the independent within subjects factors condition (pre-post feedback) and the between factor BAS (high-BAS vs. low-BAS) applied to ER, RTs and self-perception variables; while within factors condition and hemisphere side (Lat, left vs. right), and the between factor BAS were applied to alpha band and O2Hb variable. The RTs were recorded from the stimulus onset, and ER was calculated as the total number of incorrect detections out of the total trial for each category. Higher values represented increased incorrect responses. About self-perception, the increased or decreased self-perceived ranking was considered. Alpha band modulation, O2Hb and HHb were calculated for each block. For all ANOVA tests, the degrees of freedom were corrected using Greenhouse–Geisser epsilon where appropriate. *Post hoc* comparisons (contrast analyses) were applied to the data. Bonferroni test was applied for multiple comparisons.

Finally, a series of regression analyses was applied to BAS, cognitive performance (ER; RTs), self-perception, O2Hb, and alpha modulation.

A preliminary analysis was conducted with gender as independent factor, to exclude any gender effect for both behavioral and neuropsychological measures. No significant effect was found at the statistical analysis.

### ANOVA

#### ER

ANOVA indicated a significant interaction effect BAS × Cond [*F*(1,31) = 6.43, *p* ≤ 0.001, η^2^ = 0.32]. High-BAS showed an increased ER in post-feedback compared to pre-feedback condition [*F*(1,31) = 7.21, *p* ≤ 0.001, η^2^ = 0.34], whereas high-BAS did not differ from low-BAS in pre- [*F*(1,31) = 5.78, *p* ≤ 0.001, η^2^ = 0.36] or post-feedback [*F*(1,23) = 7.51, *p* ≤ 0.001, η^2^ = 0.36] (**Figure 3**). Also, no significant differences were found for low-BAS between pre- and post-feedback [*F*(1,23) = 1.09, *p* = 0.21, η^2^ = 0.12].

#### RTs

**FIGURE 4:**
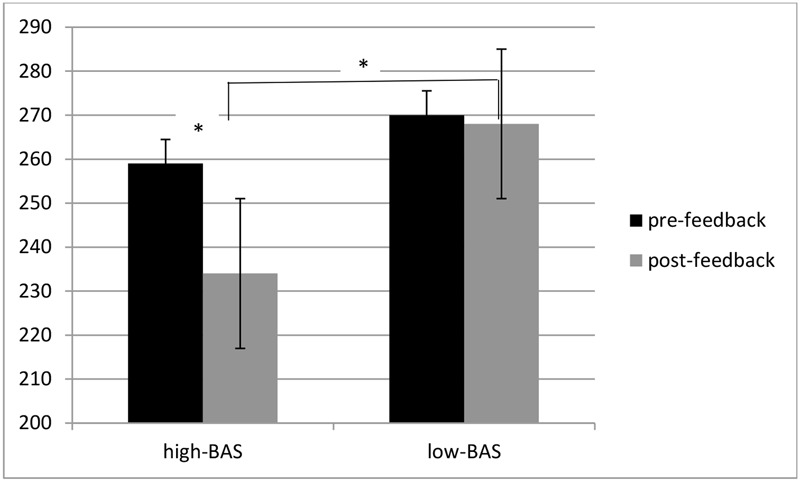
**Response times (RTs) modulation as a function of BAS and Condition.** High-BAS subjects showed decreased RTs in post-feedback compared to pre-feedback condition, and reduced RTs compared to low-BAS during post-feedback condition. Asterisk indicates significant differences.

ANOVA indicated significant main effects for Cond [*F*(1,31) = 8.23, *p* ≤ 0.001, η^2^ = 0.40], with decreased RTs during post-feedback condition; for BAS [*F*(1,31) = 6.32, *p* ≤ 0.001, η^2^ = 0.37], with decreased RTs for high-BAS; and an interaction effect BAS × Cond [*F*(1,60) = 8.11, *p* ≤ 0.001, η^2^ = 0.41]. High-BAS showed decreased RTs in post-feedback compared to pre-feedback condition [*F*(1,31) = 8.50, *p* ≤ 0.001, η^2^ = 0.40], and reduced RTs compared to low-BAS in post-feedback [*F*(1,31) = 5.31, *p* ≤ 0.001, η^2^ = 0.30]. No other comparison was statistically significant (**Figure 4**).

#### Self-Ranking

**FIGURE 5:**
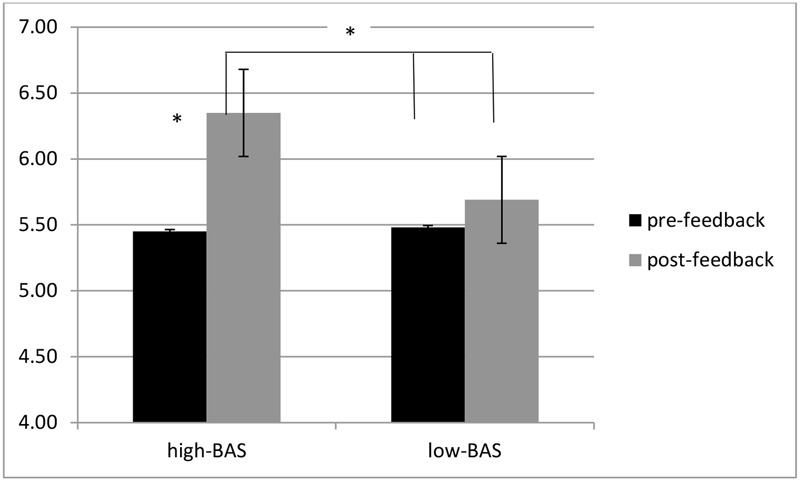
**Ranking self-perception modulation as a function of BAS and condition.** High-BAS showed higher ranking perception than low-BAS in pre-feedback and post-feedback, as well as higher ranking in post- than pre-feedback. Asterisk indicates significant differences.

About the evaluation of their ranking position in term of performance, ANOVA indicated significant interaction effects for BAS × Cond [*F*(1,60) = 7.34, *p* ≤ 0.001, η^2^ = 0.36]. Indeed high-BAS showed higher ranking perception than low-BAS in pre-feedback [*F*(1,31) = 6.55, *p* ≤ 0.001, η^2^ = 0.34] and post-feedback [*F*(1,31) = 6.90, *p* ≤ 0.001, η^2^ = 0.35]. In addition, high-BAS revealed higher ranking in post- than pre-feedback [*F*(1,31) = 7.11, *p* ≤ 0.001, η^2^ = 0.37] (**Figure 5**).

#### Alpha Band

**FIGURE 6:**
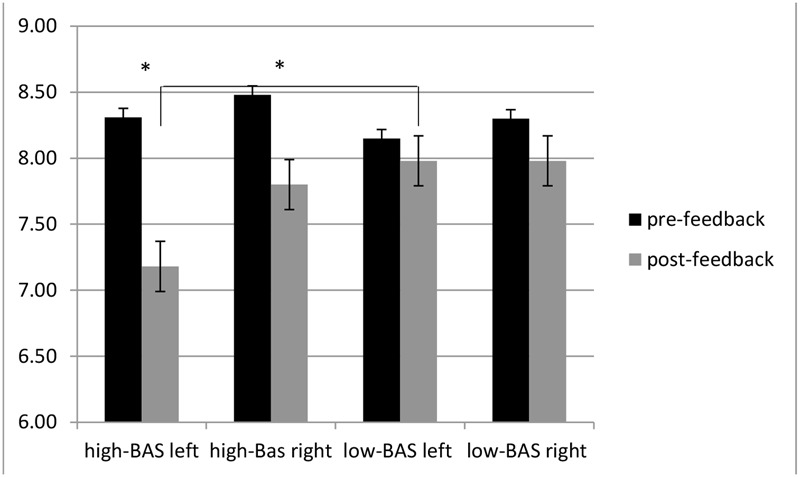
**Alpha band variation as a function of BAS and Condition.** High-BAS showed increased left response in post-feedback than pre-feedback condition and compared to low-BAS in post-feedback condition. Asterisk indicates significant differences.

ANOVA indicated significant interaction effects for Lat × Cond [*F*(1,60) = 7.50, *p* ≤ 0.001, η^2^ = 0.35], with decreased left alpha activity (increased brain response) for post-feedback compared to pre-feedback condition; BAS × Lat [*F*(1,60) = 7.37, *p* ≤ 0.001, η^2^ = 0.37], with increased left activity (reduced alpha) for high-BAS than low-BAS [*F*(1,30) = 6.98, *p* ≤ 0.001, η^2^ = 0.35]; BAS × Lat × Cond [*F*(1,119) = 8.60, *p* ≤ 0.001, η^2^ = 0.40], with increased left response for high-BAS in post-feedback than pre-feedback condition [*F*(1,30) = 5.90, *p* ≤ 0.001, η^2^ = 0.31]; and with increased left response for high-BAS than low-BAS in post-feedback condition [*F*(1,30) = 7.13, *p* ≤ 0.001, η^2^ = 0.37] (**Figure 6**).

#### fNIRS

The statistical analyses were applied to D dependent measure for O2Hb and HHb-concentration. The analysis on HHb did not reveal significant effects, and for this reason we report only results for O2Hb-values. D dependent measure was subjected to three factors (BAS × Cond × Lat) repeated measure ANOVA. The data were averaged over left (Ch1: FC3-F3; Ch2: FC3-FC1; Ch5: F1-F3; Ch6: F1-FC1) and right (Ch3: FC4-F4; Ch4: FC4-FC2; Ch7: F2-F4; Ch8: F2-FC2) channels.

**FIGURE 7:**
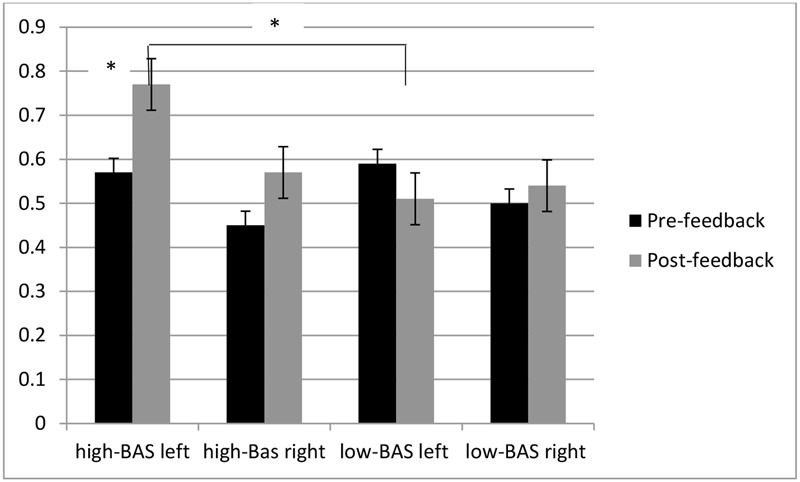
**O2Hb modulation ad a function of BAS and Condition.** High-BAS showed increased left response for in post-feedback than pre-feedback condition and compared to low-BAS in post-feedback condition. Asterisk indicates significant differences.

As shown by ANOVA, Lat × Cond was significant [*F*(1,31) = 7.12, *p* ≤ 0.001, η^2^ = 0.35] with increased left activity for post-feedback compared to pre-feedback condition; BAS × Lat [*F*(1,31) = 8.40, *p* ≤ 0.001, η^2^ = 0.40], with increased left activity for high-BAS than low-BAS [*F*(1,23) = 9.70, *p* ≤ 0.001, η^2^ = 0.45]; BAS × Lat × Cond [*F*(1,119) = 9.03, *p* ≤ 0.001, η^2^ = 0.44], with increased left response for high-BAS in post-feedback than pre-feedback condition [*F*(1,31) = 8.01, *p* ≤ 0.001, η^2^ = 0.42]; and with increased left response for high-BAS than low-BAS in post-feedback condition [*F*(1,31) = 7.77, *p* ≤ 0.001, η^2^ = 0.38] (**Figure 7**).

### Regression Analyses

**FIGURE 8:**
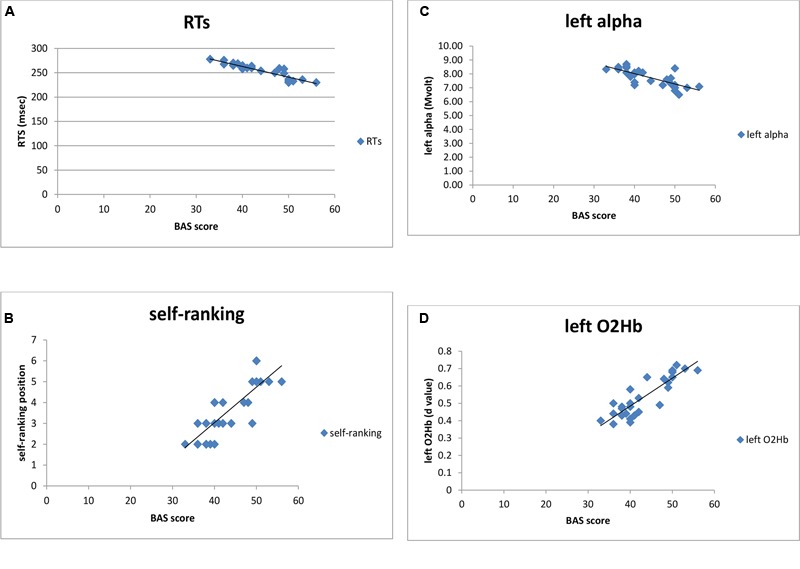
**Stepwise regression analyses, between **(A)** BAS and RTs, **(B)** BAS and self-ranking, **(C)** BAS and alpha band, **(D)** BAS and left O2Hb**.

Distinct stepwise regression analyses were performed. Predictor variables were BAS scores and the predicted variable were, respectively, self-ranking, performance (ER, RTs), alpha and O2Hb. As shown, BAS score predicted RTs (*R*^2^ = 0.26, *p* = 0.001; **Figures 8A–D**), since increased BAS was related to RTs reduction. In contrast, no significant result was found for ER (*R*^2^ = 0.09, *p* = 0.15). In addition, BAS predicted self-ranking score (*R*^2^ = 0.23, *p* = 0.001), with a positive relation between the two measures. Also, left alpha (*R*^2^ = 0.29, *p* = 0.001) and left O2Hb (*R*^2^ = 0.22, *p* = 0.001) values were predicted by BAS, as shown in **Figure 8**. Indeed BAS increased values explained alpha reduction (brain activation) within the left PFC; similarly BAS increased values explained O2Hb increasing within the left PFC.

## Discussion

The present research intended to explore the effect of personality components and brain correlates in social ranking perception during a cognitive task which included a competitive joint-action and where a performance-based feedback was provided. Specifically, brain oscillations (alpha band) and hemodynamic brain activity (O2Hb) were considered in high- and low-BAS profile subjects, to elucidate how this motivational trait component may affect subjects in formulating self-representation (ranking position) and self-improvement (cognitive performance) in an interpersonal context. Based on our research, some main results were observed. A first result was related to the systematic PFC response (reduced alpha and increased O2Hb) to social ranking perception during competition. This PFC activation was mainly found in response to reinforcing condition (positive post-feedback) induced in the subjects for their competitive joint-action. In relation to this enhanced PFC responsiveness, a specific lateralization effect was found, with increased left activity in comparison to the right one. A second main result was related to the improved performance in terms of RTs, with reduced RTs after a positive reinforce. Also, self-perception of ranking position showed to be increased in relation to post-reinforce condition. A third main result was obtained for BAS variable, with a consistent impact of higher-BAS scorings on brain activity (more left lateralized response to reinforce), cognitive performance (general improved cognitive outcomes), and self-perception (with higher ranking for high-BAS).

About the first effect, a consistent PFC contribution was detected in response to post-feedback condition which reinforced the good subjective performance compared to C’s performance. Specifically, as shown by EEG and fNIRS data, the PFC was mainly implicated when subject were informed about their better outcomes. This result is in line with previous research which suggested a main role of the VMPFC in responding to status (Karafin et al., 2004). Indeed, recent studies investigating the effect of interpersonal situations and reciprocal strategies when playing with cooperative, neutral, and non-cooperative human partners, found differential activation in the DLPFC (Suzuki et al., 2011) and activation in the superior temporal sulcus during successful adaptation to the strategies of computer agents (Haruno and Kawato, 2009). In addition, in clinical domain it was found that patients with VMPFC lesions made less use of information for their dominance judgments (Karafin et al., 2004). Moreover, De Vico Fallani et al. (2010) by using EEG hyperscanning technique have reported activation in this region during reciprocal interaction in an iterated Prisoner’s Dilemma game. Given the evolutionary prevalence and importance of social ranking and social perception in interactive contexts, where hierarchy across species and across human social groups is crucial, it is plausible that the “social” brain has specialized mechanisms for perceiving social status and joint-actions.

In parallel to variations in brain activity, a general better performance with decreased RTs was found especially when subjects perceived themselves as a better performer than the other competitor. This main result was in line with previous data, which pointed out the role of a competitive contexts to induce improved cognitive outcomes (with general decreased RTs) compared to cooperative ones, mainly when we develop a general sense of dominance and superiority than others. More interestingly, the post-feedback condition revealed concomitant and simultaneous increased PFC responsiveness, better cognitive outcomes, and a significant increased self-perception ranking. Therefore, the external evidence (although artificially induced) of a good cognitive performance produced also a consistent impact on social perception, with higher self-attribution in ranking position. In parallel it was able to induce a real higher cognitive performance compared to no-feedback condition.

We may suppose that this consistent effect was related to the impact of the perceived performance on the cognitive real performance, when this rating was compared to that of the C. Secondly, this intrinsic relation between brain, cognitive behavior, and social representation might highlight the possibility of considering the reciprocal influence of the PFC and self-perception, and of the PFC and the cognitive task, as they may be represented as two sides of the same coin. That is, a sort of “circular effect” may be adduced: from one hand, the social significance of the performance for the social hierarchy appears to be highly relevant in modulating the subjects’ performance across the task (with a consistent and parallel increasing of ranking perception and subjective performance), that is modulated by the prefrontal areas which could support the social perception process. From the other hand, the suggested increasing in cognitive outcomes may affect the self-perception of ranking position, with evident gains for the subjective representation of social status. Also, in this second case the PFC may support the reciprocal relationship between cognitive performance and social representation, reinforcing the “social value” of the prefrontal areas (Freeman et al., 2009; Marsh et al., 2009; Koslow et al., 2013; Liu et al., 2015).

At this regard the theory of social comparison processes (Festinger, 1954) suggested an important role for hierarchical rank in achieving accurate self-knowledge and self-improvement for subjects. Therefore these two components (social ranking perception; cognitive performance) may be considered as crucial components which can affect the subjective behavior, and the PFC activation could be suggested as the underlying correlate of this efficient mechanism. However, it should be noted that, in comparison to some previous research (Dötsch and Schubö, 2015), in the present study modifications in performance and self-representation were not generated when a generic positive reinforce was provided, but they were explicitly related to a vis-à-vis interactions: subjects “saw” by themselves (as indicated by the visual feedback) to be more capable than the other C. We may suggest that in the present condition subjects represented their social performance as the key point of their behavior.

In concomitance, a clear hemispheric lateralization effect was observed. As elucidated by the present data, a left lateralized cortical network within the PFC was found in concomitance to positive feedback. More generally, in this study, the left hemispheric effect was demonstrated to be prominent to explain our results, taking into account both O2Hb and EEG modulations. The fact that this cortical “unbalance” in favor of the left hemisphere in response to positive reinforcing conditions was also accompanied by a better performance and an increased social efficacy during post-feedback condition in terms of ranking attribution, may suggest an underlying link between the left cortical activity, the external social ranking representation, and the effective behavior. The specific cortical localization may suggest the consistent over-activation of the left cortical system and a concomitant predominance of this brain area in managing subjects’ cognitive behavior when they perceived to be higher in ranking. To support this interpretation, previous research demonstrated that high social power perception is indeed associated with greater left frontal brain activity compared to low social power (Boksem et al., 2012).

However, to deeply comprehend the present data we need to refer to a crucial and ampler construct, that is the role of a personality component defined as “approach attitude.”

In fact, it has been previously suggested that the frontal cortical asymmetry in favor of the left hemisphere is associated to approach motivations and with general dominance and, therefore, to BAS, together with the ability to regulate positive emotions (Davidson, 1993; Jackson et al., 2003; Urry et al., 2004; Balconi and Mazza, 2010; Harmon-Jones et al., 2010). Specifically it was shown that, based on resting intracortical activity during social threat, participants with higher resting activity in the left vs. right DLPFC cortex exhibited more adaptive, dominant, and approach-oriented responses (Koslow et al., 2013). These results suggest that social status may not be a “universally valid” and immutable phenomenon; rather, the perception of our own ranking, particularly during conditions of competition with others, may be directly and strongly related to personality approach-related components. This is in line with previous studies (Demaree, 2005), which reported that those individuals with a higher-BAS were more likely to relate to the dominant and “proactive” character in situations which were shown to induce a positive effect, whilst those with a higher BIS sensitivity were more inclined to relate to a submissive and passive character, inducing a negative effect.

More generally, as shown by the regression analysis, in the present research it was observed that personality approach attitude (high-BAS) was able to modulate brain activity (for both O2Hb and alpha band), social ranking perception, and cognitive performance. Indeed the perception of ranking was improved in post-feedback for high-BAS, with self-representation as higher in social status compared to the C. Moreover, we may describe high-BAS performance as better than low-BAS one, mainly in term of RTs, although they generally have “paid” for their RT reduction with higher ER in post-feedback. In the case of competition high-BAS had significant higher PFC responsiveness, but limited to a specific module localized within the left hemisphere, as shown by both EEG (alpha decreasing) and O2Hb (increased values). Therefore the lateralization effect was also confirmed in the case of high-BAS with higher left PFC after post-feedback reinforce.

These results are in line with some previous studies which demonstrated that high cortical left unbalance is related to approach-related conditions, with higher prevalence of high-frequency oscillations in the left than the right PFC (Balconi and Mazza, 2010). It is possible to explain this lateralization effect by pointing out that approach attitude, generally associated to increased left PFC responsiveness, is able to affect *per se* both the self-perception of efficacy and competition and, consequently, the subjects’ real performance. Indeed we may state that a more consistent approach attitude and positive motivation may support a concomitant left side hyperactivation which supports the self-representation of an increased social ranking in cooperative contexts, with an improved real cognitive performance. Nevertheless, the role of BAS was not able in absolute to explain the present results, since we had a significant generalized higher left hemispheric activity also independently from BAS contribution. In other terms, the “basic” left lateralized BAS effect due to approach attitude might not exhaustively explain the increased effect found in post-feedback condition, with higher left DLPFC activity as a consequence of positive reinforcing. We may suggest that the left PFC contribution in competition may be also intended as a specific cortical module associated to positive experiences, positive feelings, and emotional valence.

In general the relevance of BAS construct may also be related to these three levels of explanation, also integrated each other: the sense of self-efficacy; the sensitivity to the reinforcing conditions and rewarding aspects; the dominance trait. From the first perspective, this raises the possibility that our personality and our subjective comprehension of social hierarchies may interact to impact our social success and sense of well-being. At this regard, it is possible that high-BAS implicitly assessed their own (self-referential) social hierarchical status more than what low-BAS did in relation to the task they performed, with particular respect to increased social efficacy perception. It is also possible that the improved self-perception of ranking (induced by the external feedback) may have introduced a reinforcing cue able to significantly modify the behavioral performance (Chiao, 2010).

Secondly, higher-BAS subjects may be more attentive to conditions that produce a significant positive reinforce, and that reinforce the behaviors which are active in nature, ingenerating positive emotions and positive self-perception of approaching attitude (Balconi et al., 2009b), as shown by previous research which used a similar condition (Balconi and Pagani, 2014, 2015). As observed in the present research, this effect could be valid and consistent also when a specific social task is provided. Thus, in line with our previous hypotheses, we observed in higher-BAS subjects a prevalence in responding to a rewarding condition that includes a joint-action also when it is competitive in nature. It should also pointed out that, as shown by previous research, the rewarding factor may impact on behavior independently from the nature and the quality of the relationship (cooperation or competition): that is any rewarding context may be potentially usable to reinforce their sense of efficacy, improved performance, and subjective responsiveness. In the meantime, a rewarding condition may increase the sense of dominance and self-represented higher status whether the individual cooperate or compete.

Thirdly, more generally we have to consider the extent to which higher-BAS individuals are more proactive and dominant in achieving their outcomes when an interpersonal goal has to be obtained (Magee and Galinsky, 2008; Pothos and Busemeyer, 2009). By virtue of having relatively a greater proactive attitude they must rely more on their resources to meet their needs (Kraus et al., 2009). In addition, an integrative explanation of the main role exercised by BAS may be proposed taking into consideration the underlying concept of core self-evaluation (CSE, Judge et al., 1997). It refers to fundamental assessments that people make about their worthiness, competence, and capabilities (Judge et al., 2004). In this respect, CSE has been conceptualized as an indicator of high approach temperament (Judge et al., 1998), orienting individuals toward seeking positive outcomes, which subsequently influence performance and well-being. In addition CSE can be described in terms of the fundamental evaluations that people hold about themselves and their ability to control and manage the external world by forming the basis of their self-appraisals and self-efficacy.

To summarize, what about the causal relations between these multiple constructs? Indeed whereas we know that BAS and external feedback are effective in modulating the cognitive performance and the brain responsiveness, regression analyses told us that BAS construct was able to affect simultaneously the neurophysiological level (EEG and O2Hb), the ranking perception and the cognitive performance. Therefore we may suppose that personality components (dominance, sensitivity to rewarding situations and to positive feedback) can modulate the self-perception, with significant impact on the cognitive performance. At the same time, it is able to affect the cortical activity. It is probably due to the original responsiveness to the approach condition that an increased PFC responsiveness was entailed with a specific left lateralization effect. In other term, the increased perception of self-efficacy, induced by the positive external feedback, was enhanced in the case of high-BAS and it may support both the higher PFC implication and the concomitant effective improved cognitive outcomes. Therefore we may suggest a sort of “ripple reinforcing effect,” where the personality trait manage the virtuous social (ranking perception), cognitive (effective performance), and brain (prefrontal activation) responses. In addition, we found that the left side system – more related to BAS polarity – accounts for the increased performance and improved self-perception: BAS subjects showed a more intense response within the left hemisphere in the case of high reinforced competitive outcomes.

Future research should more exhaustively consider a direct comparison between cooperative and competitive tasks, to elucidate the main impact of personality components (BAS) in these two different social contexts. In addition, the distinct role of EEG and hemodynamic measures should be better tested in order to furnish a clear overview about the specificity of each of them in explaining the joint-actions. Finally, future research should provide a complete analysis of the joint-strategies used by competitors by applying specific hyperscanning method in order to consider the joint brain activities of the two actors.

## Author Contributions

MB: Planned the experiment; supervised data acquisition and data analysis; discussed the results; wrote the paper. MV: Acquired and analyzed the data; discussed the results; wrote the paper.

## Conflict of Interest Statement

The authors declare that the research was conducted in the absence of any commercial or financial relationships that could be construed as a potential conflict of interest.
